# Real-Time Optimal States Estimation with Inertial and Delayed Visual Measurements for Unmanned Aerial Vehicles

**DOI:** 10.3390/s23229074

**Published:** 2023-11-09

**Authors:** Xinxin Sun, Chi Zhang, Le Zou, Shanhong Li

**Affiliations:** 1 School of Artificial Intelligence and Big Data, Hefei University, Hefei 230601, China; sunxin@hfuu.edu.cn (X.S.);; 2 School of Advanced Manufacturing Engineering, Hefei University, Hefei 230601, China

**Keywords:** data fusion, motion estimation, inertial sensors, vision delay, UAV

## Abstract

Motion estimation is a major issue in applications of Unmanned Aerial Vehicles (UAVs). This paper proposes an entire solution to solve this issue using information from an Inertial Measurement Unit (IMU) and a monocular camera. The solution includes two steps: visual location and multisensory data fusion. In this paper, attitude information provided by the IMU is used as parameters in Kalman equations, which are different from pure visual location methods. Then, the location of the system is obtained, and it will be utilized as the observation in data fusion. Considering the multiple updating frequencies of sensors and the delay of visual observation, a multi-rate delay-compensated optimal estimator based on the Kalman filter is presented, which could fuse the information and obtain the estimation of 3D positions as well as translational speed. Additionally, the estimator was modified to minimize the computational burden, so that it could run onboard in real time. The performance of the overall solution was assessed using field experiments on a quadrotor system, compared with the estimation results of some other methods as well as the ground truth data. The results illustrate the effectiveness of the proposed method.

## 1. Introduction

There is a growing interest in autonomous aerial vehicles, which have a wide range of applications in mobile missions such as surveillance, exploration and recognition in different environments. Motion information of the vehicle, generally separated into rotation and translation, is needed to realize autonomous implementation of the system. The estimation of rotation has typically been well resolved using measurements from an onboard strap-down inertial navigation system [1]. The rotation information can be provided by a low-cost IMU [2], which is more accurate and less time consuming than pure visual algorithms. Therefore, we used the rotation information directly from the IMU and focused on the estimation of translation in this paper, including position and translational velocity.

Various kinds of sensors have been utilized in this field. The combination of GPS with gyroscopes, accelerometers, and magnetometers provided positional and velocity information as described in [3]. However, the downside is that GPS is susceptible to weather and terrain conditions, and it expends significant power. Laser range and visual-based sensors were used in [4] to obtain accurate position information, and in [5], a laser range sensor combined with visual-inertial odometry was proposed to help complete accurate positioning. However, laser range sensors also have some disadvantages, including their limited perception range and their excessive weight for UAVs. Doppler radars [6] and ultrasonic sensors [7] were chose onboard to solve this location issue. However, they are also constrained by factors such as accuracy, cost, weight, and environmental limitations. Vision sensors, due to their excellent performance in these areas, therefore have become a popular choice for obtaining motion information in the system [8].

Previous research obtaining motion information with the assistance of a visual input has been carried out in several ways. Dual cameras could reconstruct the captured environment and obtain the location of the system using a stereo computer vision algorithm. These methods were mentioned in [9,10]. However, in a micro aerial vehicle system, the weight of onboard equipment is usually hoped to be as light as possible, with the consideration of payload and cruise duration of the vehicle. Therefore, monocular cameras and related algorithms have been researched and implemented in a wide range of applications.

The optical flow method mentioned in [11,12,13] utilized pixel changes within the sequential image frames in the time domain and the correlation between adjacent frames to establish the corresponding relationship between previous and current frames, enabling the calculation of the object’s translation velocity. By integrating this velocity, the position information of the object could be obtained. The key advantage of this method is that it performs motion estimation without any requirement for knowledge about the scene. However, the position result will drift over time due to an unbounded accumulation of integration error, and the amount of computation required is enormous. 

In [14,15,16], artificial landmarks with known information were laid on the ground, and image processing technology was used to detect and extract the feature point information of the artificial landmarks. Based on this, a coordinate system transformation model was established using coordinate system transformation and camera imaging models, thereby obtaining the position information of the UAVs. However, these methods only work in certain fixed environments.

In [17,18], the feature-based methods mentioned were designed to detect and match features points between the current video frame and the reference frame. The feature matching method mentioned in [17] was designed to detect and match features across multiple frames. In contrast, ref. [18] reported a feature tracking approach that specifically matched features between adjacent frames. Once corresponding points were identified, they were used to solve a visual equation to obtain information regarding relative rotation and translation. The semi-direct monocular visual odometry (SVO) proposed in [19] combines direct methods and feature-based methods for motion estimation. This method does not require feature points to be extracted from each frame, but rather transfers feature points of the current frame from the previous frame using an optical flow approach. Feature extraction is only necessary when inserting new key frames in the mapping thread. Therefore, improved robustness and real-time performance have been achieved. Direct Sparse Odometry (DSO) [20] combines the direct method with sparse reconstruction to extract the brightest pixel positions in image sequences. By monitoring sparse pixel groups, it takes into account image generation parameters and adopts indirect monitoring procedures. It should be pointed out that DSO only works perfectly when using photometric cameras for calibration, rather than conventional cameras, which do not provide high-precision results. Since the focus of this paper is on fusion of IMU data with pure visual sensor motion estimation information, the optimization of the pure visual motion estimation algorithms is not involved in this study. 

Vision–inertial fusion methods were also studied by many researchers to estimate motion state. In [21], inertial data were used to establish motion estimation equations together with visual results, rotation and scale factor were also estimated with these equations, which was different from the method proposed in this paper.

Observing that the rotation information could also be provided by the IMU [2], there are redundant calculations here. More pairs of corresponded points are needed to solve the extra undetermined rotation variables, which could otherwise be directly obtained from the IMU. Besides extra computational complexity, another disadvantage is that wrong pairs of corresponded points will affect other correct ones when they are contained in one equation. A monocular vision algorithm alone is not able to obtain real scale but just the direction of translation; related solutions were mentioned in [22].

To obtain accurate, fast-updated and reliable states estimation of the system, position observation directly from a vision algorithm is usually fused with inertial information, normally based on expanded versions of the Kalman filter [11]. However, taking the multi-rate of the sensors and the delay of visual observation caused by hardware, wireless transmission and processing time of the vision algorithm into consideration, the classic Kalman filter model is not exactly the same as the model here and needs to be modified.

A single-rate Kalman filter with delayed measurement was researched in [23], and a solution was proposed by extrapolating the measurement. To reduce the computational burden, stable Kalman gain instead of real Kalman gain was used in the method, causing non-optimality. In [24], the residual was calculated using current measurements and past corresponding estimates, and it was then fused using normal Kalman filter update rules. However, due to the asynchronous nature between the estimate and the residual, it is not optimal.

In this article, a solution of motion estimation is introduced for an aerial vehicle with an onboard IMU and a downward-looking monocular camera fixed on it. The environment below the camera is assumed to be basically planar. The whole solution is separated into two steps.

First we presented a novel method to obtain the 3D local position from vision. Different from previous pure vision algorithms, the attitude information provided by the IMU was integrated in the vision equations as known parameters, rather than being treated as undetermined parameters.

With the assistance of a height sensor, this algorithm could obtain a measurement of the position just with a single pair of corresponded points, which makes the algorithm faster and more robust. Second, a multi-rate optimal filter is presented to fuse vision information and inertial information with consideration of the delayed measurement. Then a modification is made to control the computational complexity so that it could be implemented on an onboard micro controller.

The rest of the paper is organized as follows. Section 2 derives the state model from the dynamic model of the UAV. Section 3 presents the method obtaining location observation from measurements with a monocular camera, an IMU, and a height sensor. Section 4 describes the framework of the state estimation of the UAV. In Section 5, a real-time filter with consideration of multi-rate sensors and delayed vision observation is introduced. Experiment results are shown in Section 6 which verifies the feasibility and performance of the proposed method. Some conclusions are presented in Section 7.

## 2. Dynamic Model

**Figure 1:**
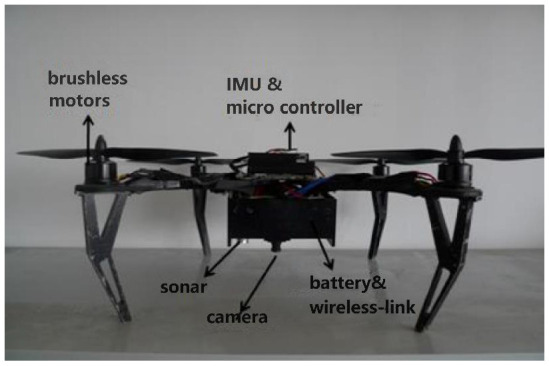
System configuration.

Our system configuration is exhibited in Figure 1. An IMU and a monocular camera with its head down are the main sensors onboard. The IMU consists of an accelerometer, a magnetometer and a gyroscope. 

Let w represent the world frame, where the $x^{w}$, $y^{w}$, and $z^{w}$ axes, respectively, correspond to the east, the north, and the vertical direction. Let b represent the body frame, where $x^{b}$, $y^{b}$, and $z^{b}$ axes adhere to the vehicle body, and the center of the body frame coincides with the system centroid.

Thus, one obtains the dynamic equation of the system motion:

(1) $$\begin{array}{l} {\dot{p}}^{w}=v^{w}\\ {\dot{v}}^{w}=a^{w} \end{array}$$

where $p^{w}=(x^{w},\;y^{w},\;z^{w})$, $v^{w}$ and $a^{w}$, respectively, represent the vehicle position, velocity, and acceleration in world frame. 

Let $R_{b}^{w}$ denote the rotation matrix from the body frame to the world frame. The vehicle acceleration in world frame could be given by:

(2) $$\begin{array}{l} a^{w}=R_{b}^{w}a^{b}\\ a^{b}=a^{m}-n_{a}-b_{a}-\vec{g} \end{array}$$

where $a^{b}$ represents the acceleration in body frame. $a^{m}$ denotes the acceleration value provided by the accelerometer adhering to the vehicle body. $n_{a}$ and $b_{a}$ denote the Gaussian noise and the bias of the accelerometer, respectively. $\vec{g}$ represents the gravity vector. $b_{a}$ could be calibrated either off-line or on-line [19]. It is not considered in this paper and the measured acceleration is regarded as non-bias. Note that because the body frame is a non-inertial frame, obtaining the complete relationship between $a^{w}$ and $a^{b}$ should take consideration of inertial acceleration, as shown in [25]. However, since the motion of the vehicle is not rigid during hovering flight, inertial acceleration is usually omitted to simplify the model in many papers, such as [22,26,27]. This simplification is adopted here and (2) is obtained. 

Substituting (2) into (1), one obtains the dynamic model of the system:

(3) $$\begin{array}{l} {\dot{p}}^{w}=v^{w}\\ {\dot{v}}^{w}=R_{b}^{w}(a^{m}-n_{a}-\vec{g}) \end{array}$$

which could be transformed into discrete form:

(4) $$\begin{array}{l} p_{k+1}^{w}=p_{k}^{w}+v_{k}^{w}\Delta t\\ v_{k+1}^{w}=v_{k}^{w}+R_{b}^{w}(a_{k}^{m}-n_{a}-\vec{g})\Delta t \end{array}$$

where Δt denotes the update cycle of the accelerometer. Let $X=(p^{w},\;v^{w})$ denote the state vector in our system, then the state model of the system could be presented as:

(5) $$\begin{array}{l} {\left[\begin{array}{c} p^{w}\\ v^{w} \end{array}\right]}_{k+1}=\left[\begin{array}{cc} 1 & \Delta t\\ 0 & 1 \end{array}\right]{\left[\begin{array}{c} p^{w}\\ v^{w} \end{array}\right]}_{k}+\\ \left[\begin{array}{c} 0\\ (R_{b}^{w}a_{k}^{m}-\vec{g})\Delta t \end{array}\right]+\left[\begin{array}{c} 0\\ -R_{b}^{w}n_{a}\Delta t \end{array}\right] \end{array}$$

## 3. Position Observation

### 3.1. Principle of Position Observation

**Figure 2:**
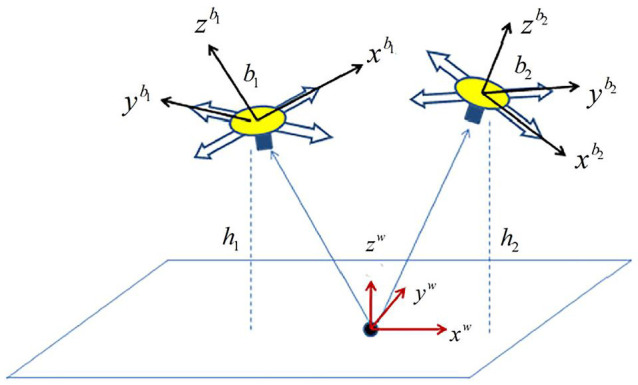
Location observation.

Figure 2 shows the environment of visual observation. The camera looks downward and captures pictures at about 30 ms per frame. A sonar sensor is fixed on the vehicle pointing downward (a barometer is used when the sonar sensor is out of range), so the height of the vehicle could be obtained.

When the vehicle moves from one location to another, the two frames will be compared in order to obtain the 3D position of the system. The first step is to detect and match the feature points between the current frame and the reference frame. This part of the algorithm has been widely studied in computer vision and significant progress has been made. We choose the Speed Up Robust Feature (SURF) algorithm in this paper because of its good qualities as suggested in [18]. 

**Figure 3:**
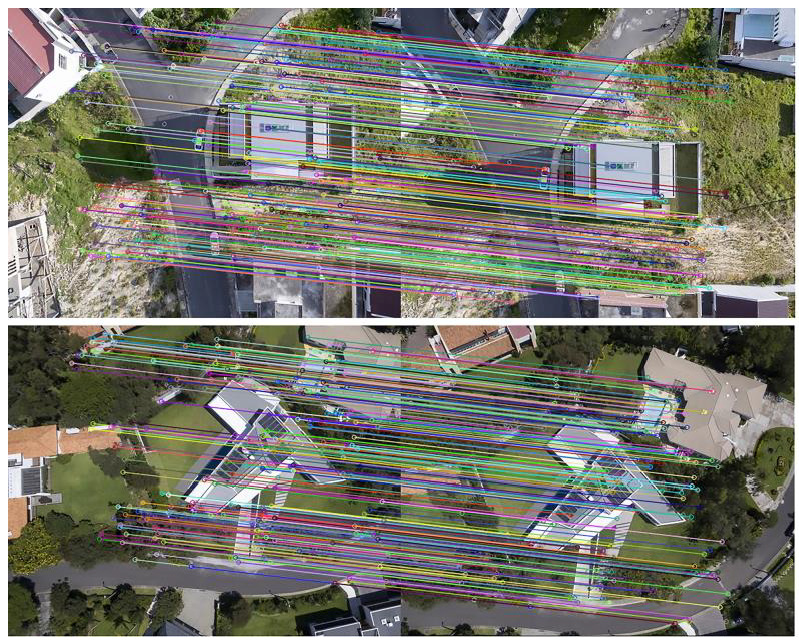
Corresponded points.

The algorithm was speeded up with a Graphic Processing Unit (GPU) to reduce the time cost. With the pairs of corresponded points obtained from the algorithm, a location observation method could be presented. In Figure 3, each pair of corresponded points is connected by a colored line. Additionally, during our hovering or small-area flight experiments, images were periodically compared with initial frames to avoid cumulative errors.

In Figure 2, the vehicle location and attitude both changed. Let $b_{1}$ and $b_{2}$ represent the body frames in two different locations, respectively. The reference video frame is captured where $b_{1}$ is located. Establish a world coordinate frame, where the center point is super positioned with one of the corresponding feature points $p_{0}^{w}={\left(x_{0}^{w},\;y_{0}^{w},\;z_{0}^{w}\right)}^{T}$. The camera frame is parallel to the body frame, with a small translation between the center points which could be obtained when the system was installed.

Transform the coordinate of $p_{0}^{w}$ in the world frame into the image frame as below:

(6) $$\begin{array}{l} s_{1}\left[\begin{array}{c} u_{1}\\ v_{1}\\ 1 \end{array}\right]=M\left[\begin{array}{cc} R_{1} & t_{1} \end{array}\right]\left[\begin{array}{c} x_{0}^{w}\\ y_{0}^{w}\\ z_{0}^{w}\\ 1 \end{array}\right]\; \end{array}$$

where $s_{1}$ is a scale factor. ${\left(u_{1},\;v_{1}\right)}^{T}$ represents a coordinate in the image frame. M could be known during calibration, which represents the intrinsic matrix of the monocular camera. $R_{1}$ indicates the rotation from the world frame to the camera frame, while $t_{1}$ denotes translation from the world frame to the camera frame. ${\left(x_{0}^{w},\;y_{0}^{w},\;z_{0}^{w}\right)}^{T}$ represents the coordinate of the feature point in the world frame, which is set as ${\left(0,\;0,\;0\right)}^{T}$, since $p_{0}^{w}$ is the center point in the world frame. Thus, the equation could be obtained as below:

(7) $$t_{1}=s_{1}M^{-1}\left[\begin{array}{c} u_{1}\\ v_{1}\\ 1 \end{array}\right]\;$$

Let $h_{1}$ indicate the measurement of the sonar sensor, and another equation could be listed to obtain the scale factor $s_{1}$:

(8) $${\left[R_{1}^{-1}t_{1}\right]}_{3}=h_{1}$$

where $R_{1}^{-1}$, the inverse of $R_{1}$, represents the rotation from the body frame to the world frame. Therefore, $R_{1}^{-1}t_{1}$ represents the camera location in the world frame. 

The camera location $\begin{array}{l} p_{c_{1}}^{w} \end{array}$ in location 1 could be obtained based on Equations (7) and (8):

(9) $$\begin{array}{l} p_{c_{1}}^{w}=R_{1}^{-1}t_{1}\; \end{array}$$

And the camera location $p_{c_{2}}^{w}$ in location 2 could also be established with the same methods shown above. Furthermore, the 3D position from location 1 to location 2 could be obtained as below:

(10) $$\begin{array}{l} p=p_{c_{2}}^{w}-p_{c_{1}}^{w} \end{array}$$

Each pair of corresponded points provides a relative position vector between the current frame and the reference frame. These results are collected together for further processing to obtain the final position observation. Mean filter and median filter are often used here. The former is fast in computation and the latter is more robust to outliers but usually requires many computational resources. Mean filter is chosen herein, but we also added a simple step to remove outliers. The location result from one single pair of corresponded points will be removed if it is quite different from the location result we obtained from the last frame.

In the observation method presented above, the rotation matrix between the world frame and the camera frame is directly obtained from the IMU. The equations are simplified so a location result could be obtained from a single pair of corresponded points independently.

Only when the vehicle is moving in a small space can two frames of images be matched. The accumulated location results obtained above should be applied to the Simultaneous Localization and Mapping (SLAM) algorithm to obtain the location results in a large space. The main idea is to accumulate small displacement with large displacement, which is discussed in [18].

### 3.2. Measurements Synchronization

Notice that the measurements from the camera and the IMU must be synchronized when they are used in the same equation, which means the attitude measurement from the IMU used in the equation must be collected at the same time as when the image is captured.

**Figure 4:**
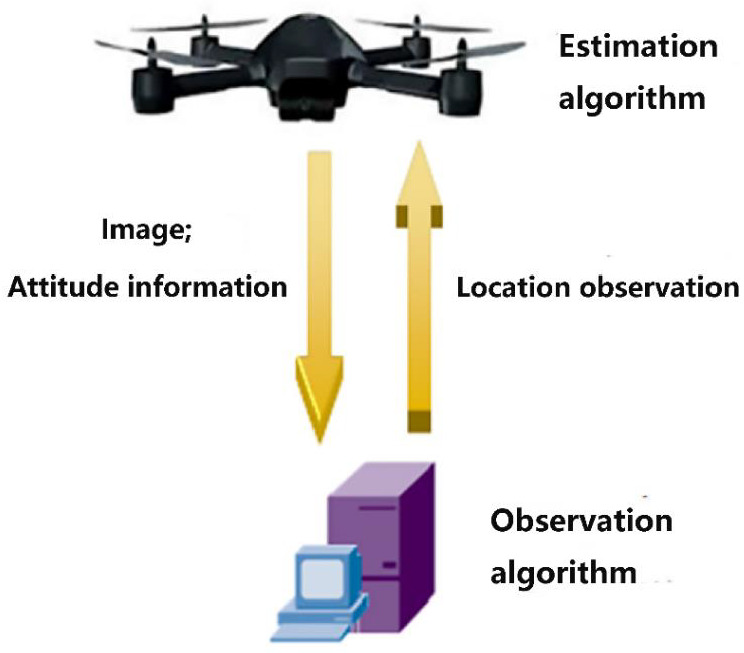
Signal transmission.

In the system, images captured with the camera onboard are transmitted through a wireless link to the ground PC computer, and so are the attitude measurements from the IMU. Then, on the PC computer, after feature detecting and matching, attitude measurements are integrated to obtain location observation, as introduced above. Then, the result of observation is transmitted back to the micro controller onboard and further fusion with inertial information will be processed there. The flow of signals is shown in Figure 4. During this cycle of transmission and processing, measurements from different sensors must be synchronized whenever they are integrated together for calculation. The delay of visual measurement mainly consists of three parts: hardware, wireless transmission and computation. We assume the delay of hardware and transmission is basically static, which is supported by experiments, as will be mentioned in Section 6. The delay of computation, mainly including feature detecting and matching, is slightly different during each processing cycle, which is approximately 90 ms to 130 ms in our system. However, the cost of computation could be measured in software each time the computation is finished and could be transmitted back onboard together with the observation result. In summary, the total delay of a visual observation result could be obtained. A data buffer is set up, both on the onboard micro controller and on the PC computer, to store IMU information during a past period of time, and the corresponded data are picked up when the visual result with known delay arrives. 

As discussed above, measurements from different sensors could be synchronized even with changing computational delay. In fact, this changing delay will not affect the observation method since the delay can be measured. However, when compensating the delay during state estimation onboard, which will be discussed in Section 5, the algorithm could be much more complex with a changing delay. Therefore, in this paper, a certain computational cycle is set in the software to make the delay fixed. It is set to longer than 130 ms to make sure it is enough for the processing of the visual algorithm most of the time in our experiment environments. The observation algorithm will return a signal of failure in case the computation is not finished during the set cycle, which has seldom happened.

## 4. Principle of State Estimation

A popular model to fuse information from multiple sensors is the Kalman filter model, which contains a state equation and an observation equation:

(11) $$\begin{array}{l} X_{k+1}=A_{k}X_{k}+B_{k}u_{k}+\Psi_{k}w_{k}\;\;state\;equation\\ Z_{k}=C_{k}X_{k}+\Phi_{k}v_{k}\;\;observation\;equation \end{array}$$

X denotes the state vector that needs to be estimated. $u_{k}$ denotes the input vector. $Z_{k}$ denotes the measurement vector. $w_{k}$ and $v_{k}$ denote the process noise and the measurement noise, respectively, which are usually considered as white Gaussian noise.

With the state model presented in Section 2 and the observation model presented in Section 3, the detailed Kalman filter model of our system could be established as:

(12) $$\left\{\begin{array}{ll} & {\left[\begin{array}{c} p^{w}\\ v^{w} \end{array}\right]}_{k+1}=\left[\begin{array}{cc} 1 & \Delta t\\ 0 & 1 \end{array}\right]{\left[\begin{array}{c} p^{w}\\ v^{w} \end{array}\right]}_{k}+\\ & \left[\begin{array}{c} 0\\ (R_{b}^{w}a_{k}^{m}-\vec{g})\Delta t \end{array}\right]+\left[\begin{array}{c} 0\\ -R_{b}^{w}n_{a}\Delta t \end{array}\right]\\ & Z_{k}=\left[\begin{array}{cc} 1 & 0 \end{array}\right]{\left[\begin{array}{c} p^{w}\\ v^{w} \end{array}\right]}_{k}+v_{k} \end{array}\right.$$

The statistical properties of $n_{a}$ are features of the accelerometer sensor, which could be found in a related hardware data sheet. Δt in our system is set to 10 ms. As mentioned in the introduction, the rotation matrix $R_{b}^{w}$ could be updated by the IMU independently. Observe that $R_{b}^{w}$ is a direction cosine matrix (which is an identity matrix) and $n_{a}$ is symmetrical in the 3 axis of the sensor; it can be proved that the covariance matrix of $-R_{b}^{w}n_{a}\Delta t$ is static, which means the covariance matrix of the processing noise $w_{k}$ is static.

In (12), some of the parameters are time invariant, while the input signal is time variant. Let:

(13) $$\begin{array}{l} A=\left[\begin{array}{cc} 1 & \Delta t\\ 0 & 1 \end{array}\right]\;C=\left[\begin{array}{cc} 1 & 0 \end{array}\right]\\ P_{w_{k}}=Q\;\;P_{v_{k}}=R\\ u_{k}=\left[\begin{array}{c} 0\\ (R_{b}^{w}a_{k}^{m}-\vec{g})\Delta t \end{array}\right]\\ B=\Psi =\Phi =I\;\; \end{array}$$

where $P_{w_{k}}$ and $P_{v_{k}}$ denote the covariance matrix of $w_{k}$ and $v_{k}$, respectively. I denotes the identity matrix. B, Ψ and Φ will be substituted directly with the identity matrix in the equations below for simplification.

**Figure 5:**
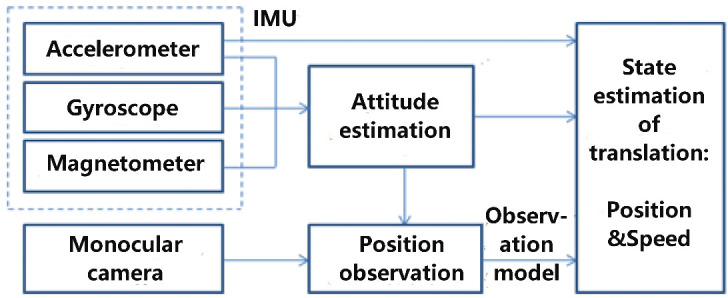
Framework of estimation.

Now we have obtained a detailed Kalman filter model of the system. The entire framework of the state estimation solution in this paper is shown in Figure 5. Data from sensors are collected, organized and processed to establish the final estimation model of the system. Notice that the attitude estimation component is solved with the method suggested in [2], and we do not present the details of the attitude estimation algorithm in this paper.

The Kalman filter is an optimal linear filter. The estimation algorithm of the Kalman filter is a recursive update algorithm, which, typically for the model in Equation (12), could be divided into two steps: 

Step 1 of the classic Kalman filter: (one-step optimal prediction) given the measurement vector ${\vec{Z}}_{k-1}={\left(Z_{1},\;Z_{2},\;\cdots ,\;Z_{k-1}\right)}^{T}$, one could obtain:

(14) $$\begin{array}{l} \hat{E}\left[X_{k}/{\vec{Z}}_{k-1}\right]=A\hat{E}\left[X_{k-1}/{\vec{Z}}_{k-1}\right]+u_{k-1}\\ P\left[X_{k}/{\vec{Z}}_{k-1}\right]=AP\left[X_{k-1}/{\vec{Z}}_{k-1}\right]A^{T}+Q \end{array}$$

where $\hat{E}\left[X_{k}/{\vec{Z}}_{k-1}\right]$ denotes the optimal estimation of $X_{k}$ with the measurement vector ${\vec{Z}}_{k-1}$. 

Step 2 of the classic Kalman filter: (optimal filtering) when a new observation $Z_{k}$ arrived:

(15) $$\hat{E}\left[X_{k}/{\vec{Z}}_{k}\right]=\hat{E}\left[X_{k}/{\vec{Z}}_{k-1}\right]+K_{kal}(Z_{k}-C\hat{E}\left[X_{k}/{\vec{Z}}_{k-1}\right])$$

where $K_{kal}$ is called the Kalman gain and could be calculated with $P[X_{k}/{\vec{Z}}_{k-1}]$, Q and R.

## 5. Delay Compensation

### 5.1. Compensation Algorithm

**Figure 6:**
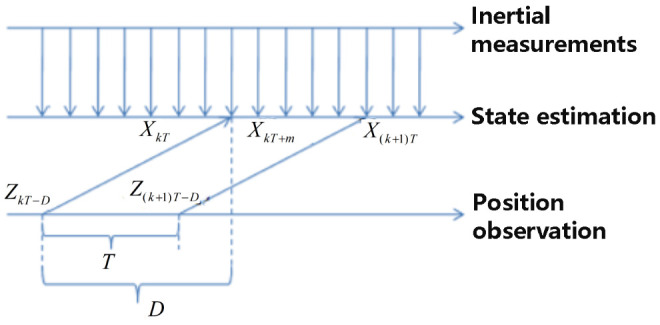
Signal sequence.

The Kalman estimation model is established in Section 4. However, the real estimation model herein is different from the classic Kalman model because the observation delay and multiple updating frequencies of sensors need to be considered, as mentioned in Section 3.2. The signal sequence in the real estimation model is shown in Figure 6.

The update cycle of the inertial measurements as the same as the control cycle of the system is 10 ms, which will be used as a unit of time in the discrete analysis. Record the update cycle of the observation as T and the delay of the observation as D, which could be tested through experiments. T is mainly caused by software computation on the PC computer, while D is caused by hardware and wireless transmission, besides software computation. Imaging that every time a cycle of computation (which is T) on the PC computer is finished and an observation result is obtained, the algorithms go on picking up the newest image just transmitted from onboard for the next cycle of computation, but the newest image already has a delay caused by hardware and wireless transmission (which is D−T). So, it could be understood that the delay of the observation is larger than the update cycle of the observation, and they need to be considered separately.

Assume that the first time we obtained an observation is at time T. Then, the observations are only obtained at the times multiple of T, and the real time at which they are measured is at time kT−D, where k≥1. The subscript of times is allowed to be negative because it is just a mark of sequence.

Record ${\vec{Z}}^{k}=\{Z_{T-D},\;\cdots ,\;Z_{kT-D}\}$ as the sequence of the first k observations we obtained.

The two steps (14) and (15) of the Kalman filter as well as a corollary of (14) will be used to obtain the optimal estimation of the state in the system with the delay of observation and the multiple updating frequency of sensors.

**Corollary 1.**  multi-step optimal prediction

(16) $$\begin{array}{l} \hat{E}\left[X_{s}/{\vec{Z}}^{k}\right]=A_{s,\;kT-D}\hat{E}\left[X_{kT-D}/{\vec{Z}}^{k}\right]+\sum_{i=kT-D}^{s-1}A_{s,\;i+1}u_{i}\\ P\left[X_{s}/{\vec{Z}}^{k}\right]=A_{s,\;kT-D}P\left[X_{kT-D}/{\vec{Z}}^{k}\right]A_{s,\;kT-D}^{T}+\sum_{i=kT-D}^{s-1}A_{s,\;i+1}QA_{s,\;i+1}^{T} \end{array}$$

*where:*

$$\begin{array}{l} A_{s,\;kT-D}=\prod_{i=kT-D}^{s-1}A_{i}=A^{s-kT-D}\\ s>kT-D \end{array}$$

*Equation (16) presents that the multi-step optimal prediction from time *kT−D* to time *s* could be obtained given the optimal estimation at time *kT−D* and the input information during the period from *kT−D* to *s*. The corollary has been detailed, derived in* [28] *with the orthogonality principle. With (14), (15) and (16) presented above, the algorithm of the state estimation with the delay observation could be presented.*

As shown in Figure 6, a new observation arrived at the time kT, which is actually measured at the time kT−D. The whole sequence of observations we have obtained during the time kT+m (0≤m<T) does not change until a new observation arrives at the time (k+1)T. The optimal linear estimation of the state during the time kT+m (0≤m<T) could be expressed as:

(17) $$\begin{array}{l} \hat{E}[X_{kT+m}/{\vec{Z}}^{k}]\;\;\\ P[X_{kT+m}/{\vec{Z}}^{k}]\\ 0\leq m<T \end{array}$$

where $\hat{E}[X_{kT+m}/{\vec{Z}}^{k}]$ denotes the optimal linear estimation of X with certain numbers of observation ${\vec{Z}}^{k}$. $P[X_{kT+m}/{\vec{Z}}^{k}]$ denotes the covariance matrix of the estimation.

Compensation algorithm: 

Initial conditions: $\hat{E}[X_{kT-D}/{\vec{Z}}^{k-1}]$ and $P[X_{kT-D}/{\vec{Z}}^{k-1}]\;$ are known. 

**Case 1:** new observation arrived at t=kT (m=0), which is actually measured at t=kT−D.

**Step 1:** optimal filtering at the time kT−D with new observation $Z_{k}$, using (15):

(18) $$\begin{array}{c} \hat{E}[X_{kT-D}/{\vec{Z}}^{k-1}]\\ P[X_{kT-D}/{\vec{Z}}^{k-1}] \end{array}\overset{\left(15\right)}{\to }\begin{array}{c} \hat{E}[X_{kT-D}/{\vec{Z}}^{k}]\\ P[X_{kT-D}/{\vec{Z}}^{k}] \end{array}$$

**Step 2:** Multi-step optimal prediction from the time kT−D to (k+1)T−D, using (16). This prediction result will be used as the initial conditions next time when a new observation arrives.

(19) $$\begin{array}{c} \hat{E}[X_{kT-D}/{\vec{Z}}^{k}]\\ P[X_{kT-D}/{\vec{Z}}^{k}] \end{array}\overset{\left(16\right)}{\to }\begin{array}{c} \hat{E}[X_{(k+1)T-D}/{\vec{Z}}^{k}]\\ P[X_{(k+1)T-D}/{\vec{Z}}^{k}] \end{array}$$

**Step 3:** Multi-step optimal prediction from the time kT−D to kT, using (16). This prediction result will be used as the current optimal estimation result.

(20) $$\begin{array}{c} \hat{E}[X_{kT-D}/{\vec{Z}}^{k}]\\ P[X_{kT-D}/{\vec{Z}}^{k}] \end{array}\overset{\left(16\right)}{\to }\begin{array}{c} \hat{E}[X_{kT}/{\vec{Z}}^{k}]\\ P[X_{kT}/{\vec{Z}}^{k}] \end{array}$$

**Case 2:** no new observation arrived (0<m<T)

**Step 1:** One-step optimal prediction from the time kT+m−1 to kT+m, using (14).

This prediction result will be used as the current optimal states estimation.

(21) $$\begin{array}{c} \hat{E}[X_{kT+m-1}/{\vec{Z}}^{k}]\\ P[X_{kT+m-1}/{\vec{Z}}^{k}] \end{array}\overset{\left(14\right)}{\to }\begin{array}{c} \hat{E}[X_{kT+m}/{\vec{Z}}^{k}]\\ P[X_{kT+m}/{\vec{Z}}^{k}] \end{array}$$

When a new observation arrives at time (k+1)T, the estimation algorithm could go on for the next cycle with the new initial conditions updated in Case 1 (Step 2) of the last cycle.

In every step of the update algorithm presented above, an optimal linear estimation result is given using all the observations obtained so far. Therefore, the update algorithm presented above is an optimal estimation algorithm.

In case of a dropout of the observation, as mentioned in Section 3.2, the observation algorithm will return a signal of failure. Therefore, in Case 1 (Step 1) a one-step prediction, the same as in Case 2 (Step 1), will be processed, instead of the filtering as usual. The effect of occasional dropouts could be reduced with the prediction, especially when the input signals are accurate.

The estimation result with dropouts of observation will be shown in Section 6.

### 5.2. Optimization of Compensation Algorithm

Because the estimation algorithm will be processed on the onboard micro controller, its computational complexity needs to be controlled. In the update algorithm presented above, Case 1 (Step 1) and Case 2 (Step 1) just use Equations (14) and (15) in the classic Kalman filter; thus the computational burden is acceptable. However, in Case 1 (Step 2) and Case 1 (Step 3), multi-step optimal prediction is processed. As shown in (16), there are two terms in the equation of the multi-step optimal prediction. The coefficient of the first term is static because A is static, so it is easy to calculate. However, the second term is a summary of the time-variant input signals, which will cause heavy computational burden if it is summarized in one control cycle when needed, e.g., at the time kT. In this paper, the computational burden is reduced with an iterative update method of this summary term. Let $U_{k}(n)$ denote this term which is defined as the “input compensation term”:

(22) $$U_{k}(n)=\sum_{i=k-n}^{k-1}A_{k,\;i+1}u_{i}$$

With a buffer of the input signals, $U_{k}(n)$ could be updated in every control cycle and could be used whenever needed without complex computation.

(23) $$U_{k+1}(n)=U_{k}(n)+u_{k}-A^{n-1}u_{k-n}$$

In Case 1 (Step 2) and Case 1 (Step 3), the input compensation term is $U_{(k+1)T-D}(T)$ and $U_{kT}(D)$, respectively. So the length of the buffer of the input signals is set to D (because D>T), which means all the input signals during the last D period are stored in a buffer used for the updating of the input compensation term.

Optimization of compensation algorithm: in the update algorithm presented in Section 5.1, two extra initial conditions need to be added:

(24) $$U_{(k+1)T-D}(T)\;\;U_{kT}(D)$$

And in every step of the algorithm, these two initial conditions should be updated using (25):

(25) $$\begin{array}{l} U_{(k+1)T+m-D}(T)=U_{(k+1)T+m-1-D}(T)+u_{(k+1)T+m-1-D}-A^{T-1}u_{kT+m-1-D}\\ U_{kT+m}(D)=U_{kT+m-1}(D)+u_{kT+m-1}-A^{D-1}u_{kT+m-1-D} \end{array}$$

These two input compensation terms are updated every step to minimize the computational complexity. They will be used every time when a new observation arrives and the multi-step optimal prediction in (16) needs to be executed.

## 6. Experiment

### Platform and Environment

A quadrotor vehicle is set up for the field experiment, with the necessary sensors mentioned above. An ATmega2560 micro controller (Atmel, SAN Jose, America) is used as the processor onboard. An MPU-6000 (TDK InvenSense, Sunnyvale, America) integrated 6-axis device which combines a 3-axis gyroscope and a 3-axis accelerometer is used to collect inertial information. Images captured with the downward-looking camera are transmitted through a wireless link to the ground PC computer for location and then observation results are transmitted back onboard for states estimation. Inertial information onboard is updated at 100 Hz. The cycle of the state estimation and control onboard is set to 100 Hz too.

The visual algorithm on the ground PC computer costs 90 ms to 130 ms, with a limit of the number of matching points utilized in our experiment, so the cycle of the algorithm, which is mentioned as T in Section 5, is set to 160 ms. An off-line method with an LED was used to test the delay caused by hardware and wireless transmission, which is mentioned as D−T in Section 5. The micro controller onboard sends a signal to light up the LED, which is monitored with the camera and transmitted to the PC computer. Then an algorithm on the PC computer is used to test if the LED is light and sends a signal back onboard if so, which costs very little computational time. Through a comparison of the occurrence time of the two signals, the micro controller could obtain the delay through the whole cycle. With this test, the delay D could be measured accurately, which is measured as 200 ms in our experiment.

An independent, motion-tracking system consisting of several fixed cameras is installed to monitor the motion states of the system. The results of the motion estimation in this assistant system are more accurate than those of the onboard system because heavy HD cameras could be used here, and the cameras do not suffer the dynamic of the vehicle. After off-line processing, including low-pass filtering and outlier removal, the result was used as the ground truth data for the performance verification of the solution proposed in this paper.

The field experiment was carried out under the monitoring of the fixed motion-tracking system. The height of the vehicle was obtained with a downward-pointing sonar sensor, as mentioned in Section 3.1. The estimation results in the *x*-axis and *y*-axis, including position and velocity, are compared with the ground truth data. The estimation results from the two other online methods without compensation of the delay were given for comparison too.

**Figure 7:**
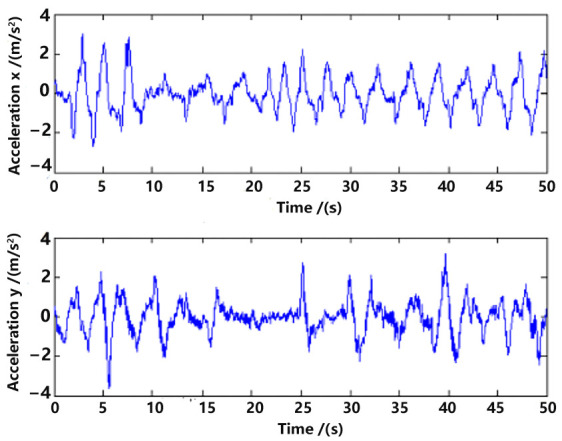
Acceleration measurement.

Raw measurements of the acceleration in the world frame ($R_{b}^{w}a_{k}^{m}$) are shown in Figure 7, which will be used in the state model (5) for estimation.

**Figure 8:**
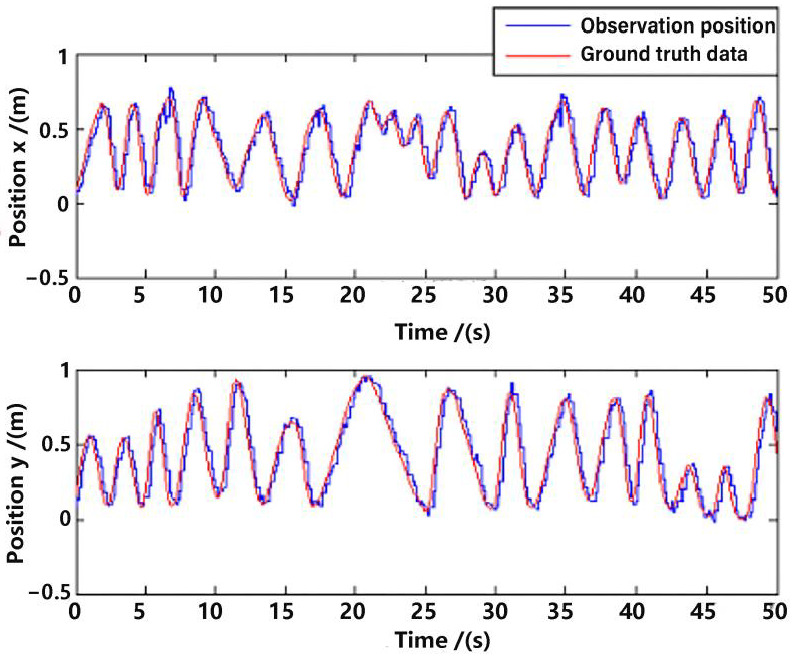
Position observation.

**Figure 9:**
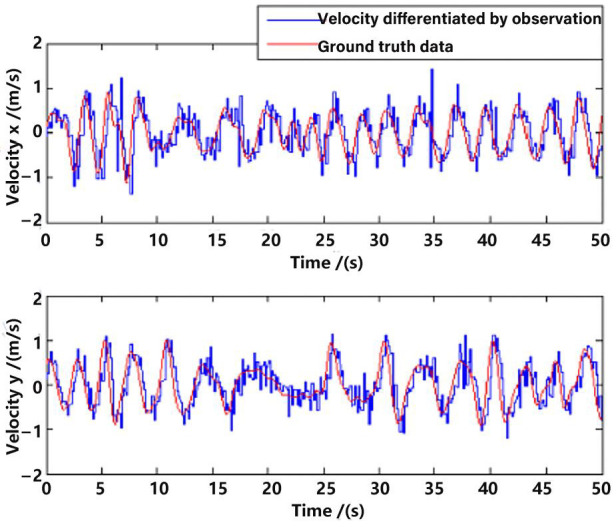
Velocity differentiated by observation.

Position observation in the *x*-axis and *y*-axis from the visual method are shown in Figure 8, with a comparison to the ground truth position data. Evidently, there is a delay between the observation and the ground truth data, which is approximately 200 ms. Ignoring the delay, the error of the observation position is not very big, which proves the feasibility of the visual observation methods in Section 3. However, if one directly differentiates the observation position data for velocity, the error will be enlarged, which is shown in Figure 9. The velocity is a very important state for the control of the UAV [16,22]; that is why multi-sensor data fusion is essential in state estimation of the UAV.

**Figure 10:**
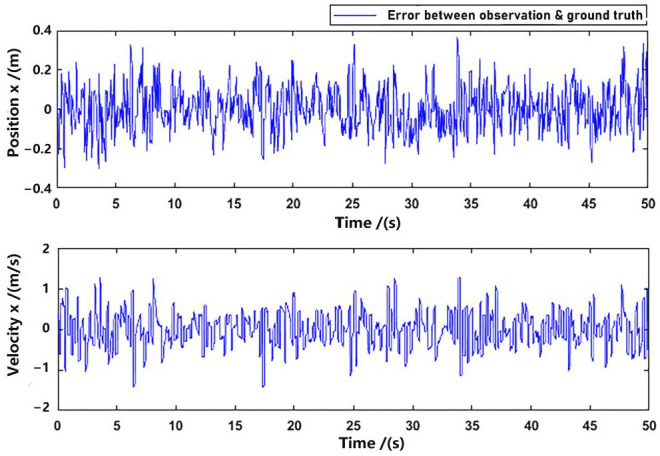
Difference between ground truth and observation (*x*-axis).

To illustrate the difference between ground truth and observation more clearly, we have subtracted the data of Figure 8 and Figure 9 (only in *x*-axis), and presented the error plot in Figure 10.

Three comparative experiments have been carried out to validate the performance of the proposed method. In the first comparative experiment, the delay of the observation was not considered, and a classic Kalman filter was directly implemented. In the second experiment, the delay of the observation was taken into consideration, so the observation data were fused with the corresponded inertial data in the data buffer, but there was no compensation in this method. In the third experiment, we compared our results with VINS-Mono (thanks to the author for open sourcing code). 

**Figure 11:**
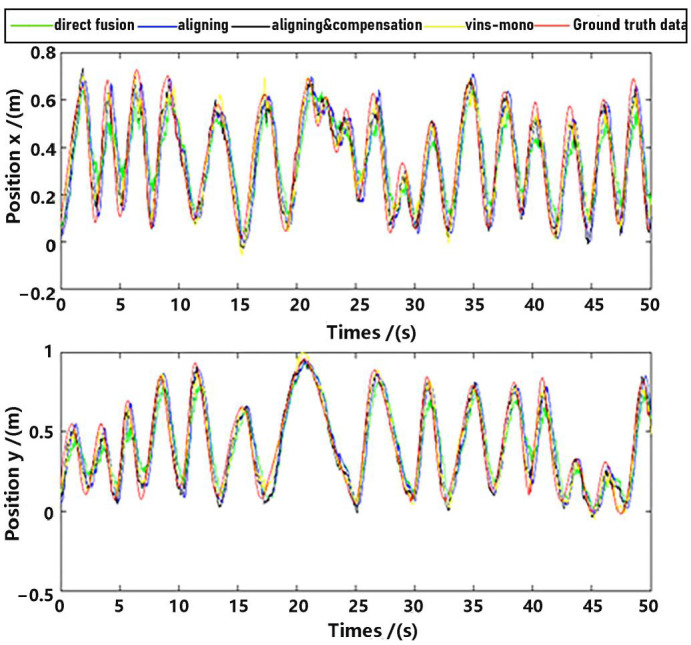
Position estimation.

**Figure 12:**
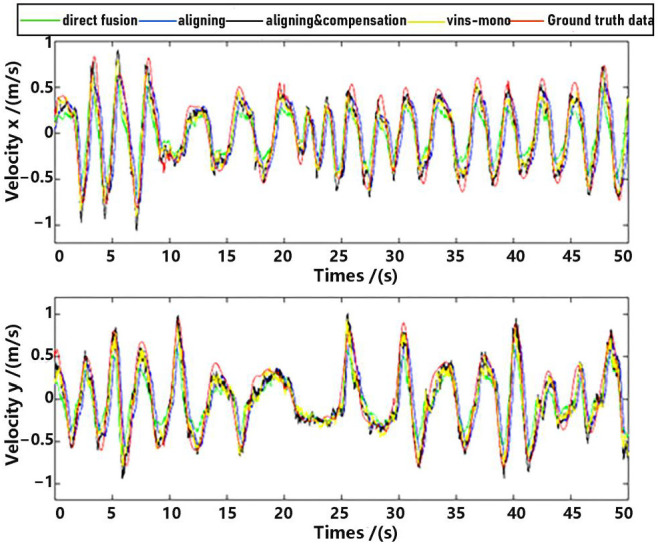
Velocity estimation.

Observe that in the method proposed in this paper, data aligning and delay compensation were both executed, so the second method actually realizes part of the function of the proposed method. Performance on the whole for methods as well as the ground truth data were shown in Figure 11 and Figure 12, where “direct fusion”, “aligning”, “vins-mono” and “aligning & compensation” denote the first comparative method, the second comparative method, VINS-Mono method and our method, respectively. Figure 11 presents the position estimation results and the latter picture presents the velocity estimation results.

**Figure 13:**
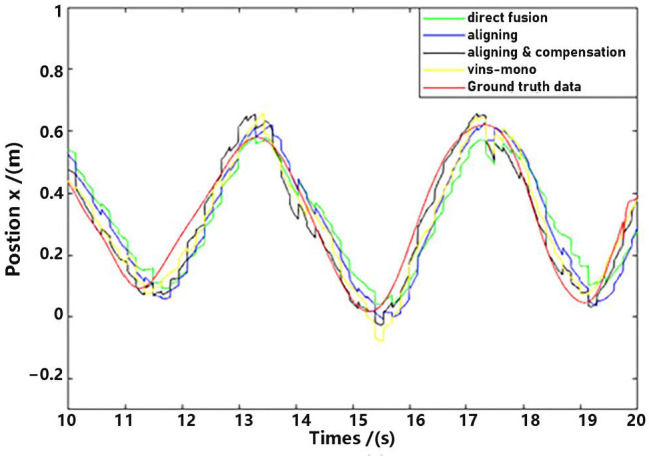
Position estimation (part).

**Figure 14:**
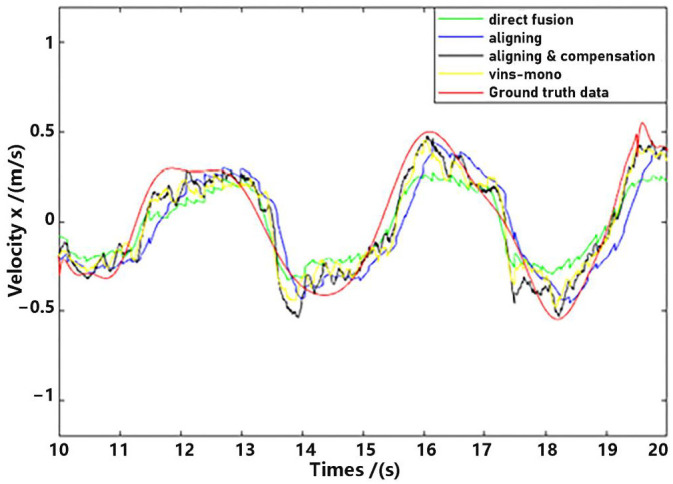
Velocity estimation (part).

For detailed performance analysis, we intercepted part of Figure 11 and Figure 12 (just in the *x*-axis) and enlarged the pictures, which are shown in Figure 13 and Figure 14.

As shown in Figure 13 and Figure 14, there is a big error between the “direct fusion” estimation and the ground truth data, especially in the peaks and valleys. The reason for this phenomenon is that the observation data (position) and the input data (inertial measurements) are not aligned, and their effects on the estimation near the extreme value could be sign opposite, so the estimation could not reach to the true extreme value. There is an obvious delay between the “aligning” estimation and the ground truth data, which is expected because no delay compensation step was executed. The results of the “aligning and compensation” estimation, compared to the former two methods, have good performance both in accuracy and delay compensation.

The experimental results also indicate that the proposed method of our paper obtains better accuracy results than the VINS-Mono methods. It is probably because we directly used inertial sensors to estimate attitude, which are more stable and accurate.

**Figure 15:**
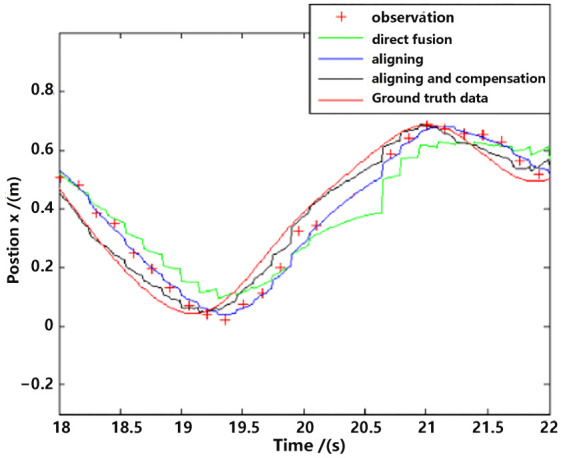
Position estimation with dropout.

**Figure 16:**
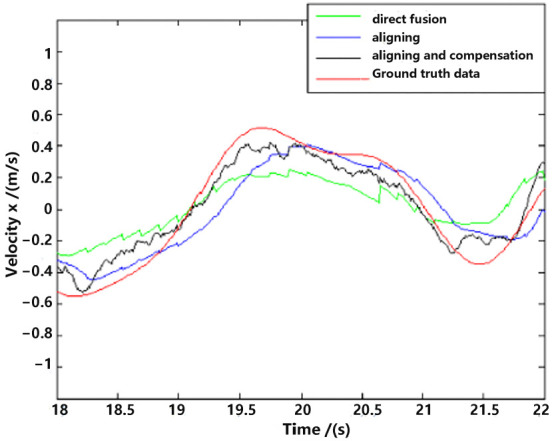
Velocity estimation with dropout.

Observation from the visual method occasionally drops out because of either hardware or software problems. The estimator proposed herein is tolerant of dropouts within a period, which is one of the main advantages of multi-sensor data fusion. In the period when observations are faulty, the prediction step is repetitively executed with the state equation and inertial measurements, as mentioned in Section 5.1, which provides acceptable estimation results. The more accurate the inertial measurements are, the slower the estimation results will diverge. One part of estimation from Figure 11 and Figure 12 with dropouts of observation is shown in Figure 15 and Figure 16.

There are three continuous dropouts of observation between 20.11 s and 20.71 s, which means there are no observation data that could be used in 600 ms. However, as shown in Figure 15 and Figure 16, the results of the estimation were affected but remained in an acceptable range.

Table 1 shows the good performance of the proposed method (estimation with data aligning and delay compensation) in both position estimation and velocity estimation. The main disadvantage of the raw observation data is that there is a big error in velocity, as mentioned and shown in Figure 9. The “Aligning” estimation method outperformed the “direct fusion” estimation method, and the performance of the proposed “aligning and compensation” method improved observably compared to these two methods.

Table 1 also shows the root-mean-square error of the results of different methods (we have collected experimental data for 2 h under various lighting conditions and background environments), which presents the performance of these estimation methods.

We also calculated the standard deviations of the errors between our method and the true values, as well as the standard deviations of the errors between vins-mono and the true values, which are shown in Table 2. We added a significance test by comparing these standard deviations with the root-mean-square errors shown in Table 1. It is evident that the difference in root-mean-square errors between the two methods is greater than that in the standard deviations. This indicates that the difference between their mean errors is not simply due to fluctuations within the data, and our method has indeed improved the accuracy of motion estimation.

To investigate the relationship between experimental results and flight speed, we conducted error analysis on the experimental results, categorizing them into different speed ranges. The results are presented in Table 3. Also, to investigate the impact of latency on fusion results, we artificially adjusted the latency of the visual data during offline data processing and summarized the error statistics of the motion estimation results under different latencies in Table 4.

Table 3 shows that the statistical error of motion estimation results remains basically unchanged at different flight speeds. This is consistent with theoretical analysis as different flight speeds do not affect the accuracy of vision data and IMU data, as well as the frequency and delay of data fusion.

Table 4 shows that as the delay increases, the estimation errors of position and velocity rapidly increase. Evidently, this is due to the accumulation of errors that cannot be corrected in a timely manner as the observation data delay increases.

## 7. Conclusions

An entire solution for the estimation of the motion of a UAV is proposed in this paper, including a vision-aided observation method and a multi-rate delay-compensated data fusion method. The observation method utilized the inertial information from the IMU to simplify the computation. The data fusion method provided real-time optimal estimation results in the condition of visual delay and multiple update frequency of sensors. The performance of the observation method has been verified with a comparison with the ground truth data, and the performance of the estimation method has been verified with a comparison with the ground truth data as well as two other estimation methods. 

Due to the requirement of image feature matching for visual localization in this method, it may fail when there are insufficient features in the background image, which can be caused by dark lighting or a smooth environment. The whole process including the visual algorithm will be put onboard in our future work. Additionally, since the visual noise in a practical environment is not static and could be affected by many factors, e.g., flight height, illumination and camera quality, another possible idea for future research is to take variant visual noise into consideration and provide adaptive estimation results. 

## Figures and Tables

**Table 1:** Root-mean-square error of the results of different methods.

Method	$P_{x}$ (m)	$P_{y}$ (m)	$V_{x}$ (m/s)	$V_{y}$ (m/s)
Raw observation	0.0897	0.0992	0.3784	0.3488
Direct estimation	0.1046	0.1207	0.2399	0.2530
Estimation with data Aligning	0.0829	0.0985	0.1843	0.1951
Estimation with data Aligning and delay compensation	0.0361	0.0434	0.1347	0.1452
VINS-Mono	0.0389	0.0463	0.1398	0.1507

**Table 2:** Standard deviations of the results of different methods.

Method	$P_{x}$ (m)	$P_{y}$ (m)	$V_{x}$ (m/s)	$V_{y}$(m/s)
Estimation with data Aligning and delay compensation	0.0025	0.0023	0.0048	0.0046
VINS-Mono	0.0024	0.0027	0.0042	0.0041

**Table 3:** Root-mean-square error of the results (aligning and delay compensation) of different velocity.

Velocity Range (m/s)	[0, 0.2)	[0.2, 0.4)	[0.4, 0.6)	[0.6, 0.8)	[0.8, 1]
$\mathrm{Error}\;\mathrm{of}\;P_{x}$ (m)	0.0356	0.0365	0.0369	0.0349	0.0371
$\mathrm{Error}\;\mathrm{of}\;V_{x}$ (m/s)	0.1344	0.1353	0.1355	0.1332	0.1358

**Table 4:** Root-mean-square error of the results (aligning and delay compensation) of different delay.

Velocity Range (m/s)	150 ms	200 ms	250 ms	300 ms	400 ms
$\mathrm{Error}\;\mathrm{of}\;P_{x}$ (m)	0.0361	0.0455	0.0689	0.0942	0.1468
$\mathrm{Error}\;\mathrm{of}\;V_{x}$ (m/s)	0.1347	0.1713	0.2358	0.2877	0.3514

## Data Availability

Data are contained within the article.

## References

[1] Jiang W., Li Y., Rizos C. (2015). Optimal Data Fusion Algorithm for Navigation Using Triple Integration of PPP-GNSS, INS, and Terrestrial Ranging System. IEEE Sens. J..

[2] Ahmed H., Tahir M. (2017). Accurate Attitude Estimation of a Moving Land Vehicle Using Low-Cost MEMS IMU Sensors. J. Turbul..

[3] Gu Y., Gross J.N., Rhudy M.B., Lassak K. (2016). A Fault-Tolerant Multiple Sensor Fusion Approach Applied to UAV Attitude Estimation. Int. J. Aerosp. Eng..

[4] Guerra E., Munguía R., Grau A. (2018). UAV Visual and Laser Sensors Fusion for Detection and Positioning in Industrial Applications. Sensors.

[5] Hao Y., He M., Liu Y., Liu J., Meng Z. (2023). Range–Visual–Inertial Odometry with Coarse-to-Fine Image Registration Fusion for UAV Localization. Drones.

[6] Whitcomb L., Yoerger D., Singh H. (1999). Advances in doppler-based navigation of underwater robotic vehicles. Proceedings of the International Conference on Robotics and Automation.

[7] Zhao H., Wang Z.Y. (2012). Motion measurement using inertial sensors, ultrasonic sensors, and magnetometers with extended kalman filter for data fusion. IEEE Sens. J..

[8] Stuckey H., Al-Radaideh A., Sun L., Tang W. (2022). A Spatial Localization and Attitude Estimation System for Unmanned Aerial Vehicles Using a Single Dynamic Vision Sensor. IEEE Sens. J..

[9] Moore R.J., Thurrowgood S., Bland D., Soccol D., Srinivasan M.V. (2009). A stereo vision system for UAV guidance. Proceedings of the IEEE/RSJ International Conference on Intelligent Robots and Systems.

[10] Boucheloukh A., Boudjema F., Abdelkrim N., Demim F., Yacef F. (2022). UAV navigation based on adaptive fuzzy backstepping controller using visual odometry. Int. J. Model. Simul..

[11] Zou Y., Xia K., He W. (2022). Adaptive Fault-Tolerant Distributed Formation Control of Clustered Vertical Takeoff and Landing UAVs. IEEE Trans. Aerosp. Electron. Syst..

[12] Herisse B., Hamel T., Mahony R. (2012). Landing a VTOL unmanned aerial vehicle on a movingplatform using optical flow. IEEE Trans. Robot..

[13] Grabe V., Bülthoff H.H., Scaramuzza D., Giordano P.R. (2015). Nonlinear ego-motion estimation from optical flow for online control of a quadrotor UAV. Int. J. Robot. Res..

[14] Eberli D., Scaramuzza D., Weiss S. (2011). Vision based position control for MAVs using onesingle circular landmark. J. Intell. Robot. Syst..

[15] Bošnak M., Matko D., Blažič S. (2012). Quadrocopter hovering using position-estimation information from inertial sensors and a high-delay video system. J. Intell. Robot. Syst..

[16] Garcia L.R., Dzul A., Lozano R. (2012). Hovering quad-rotor control: A comparison of nonlinearcontrollers using visual feedback. IEEE Trans. Aerosp. Electron. Syst..

[17] Klose S., Wang J., Achtelik M. (2010). Markerless, vision-assisted flight control of a quadrocopter. Proceedings of the IEEE/RSJ International Conference on Intelligent Robots and Systems.

[18] He M., Zhu C., Huang Q., Ren B., Liu J. (2020). A review of monocular visual odometry. Vis. Comput..

[19] Forster C., Pizzoli M., Scaramuzza D. (2014). SVO: Fast semi-direct monocular visual odometry. Proceedings of the IEEE International Conference on Robotics & Automation.

[20] Engel J., Koltun V., Cremers D. (2017). Direct sparse odometry. IEEE Trans. Pattern Anal. Mach. Intell..

[21] Qin T., Li P., Shen S. (2017). VINS-Mono: A Robust and Versatile Monocular Visual-Inertial State Estimator. IEEE Trans. Robot..

[22] Zhang B., Xu C. (2019). Research on UAV Attitude Data Fusion Algorithm Based on Quaternion Gradient Descent. Proceedings of the International Conference on Communications, Information System and Computer Engineering Guilin University of Electronic Technology.

[23] Salahshoor K., Mosallaei M. (2008). Process Fault Monitoring Using Data Fusion Based on Extended Kalman Filter Incorporated with Time-Delayed Measurements. IFAC Proc. Vol..

[24] Bourgeois F., Kneip L., Weiss S., Siegwart R. (2010). Delay and dropout tolerant state estimation for MAVs. Proceedings of the 12th International Symposium on Experimental Robotics.

[25] Cheviron T., Hamel T., Mahony R., Baldwin G. (2007). Robust nonlinear fusion of inertial and visual data for position, velocity and attitude estimation of UAV. Proceedings of the IEEE International Conference on Robotics and Automation.

[26] Krznar M., Kotarski D., Pavković D., Piljek P. (2020). Propeller speed estimation for unmanned aerial vehicles using Kalman filtering. Int. J. Autom. Control..

[27] Chen H., He X., Qing L., Wu Y., Ren C., Sheriff R.E., Zhu C. (2022). Real-world single image super-resolution: A brief review. Inf. Fusion.

[28] Fu M.Y., Deng Z.H., Yan L.P. (2010). The Kalman Filter Theory and It’s Applicatioin in Navigation Systems.

